# An integrated metabolomic and transcriptomic analysis reveals the dynamic changes of key metabolites and flavor formation over Tieguanyin oolong tea production

**DOI:** 10.1016/j.fochx.2023.100952

**Published:** 2023-10-21

**Authors:** Chenxue Li, Jiaqi Lin, Qingcai Hu, Yun Sun, Liangyu Wu

**Affiliations:** College of Horticulture, Fujian Agriculture and Forestry University, Fuzhou 350002, People’s Republic of China

**Keywords:** Tieguanyin tea, Metabolomics, Transcriptomics, Enzymatic-catalyzed phase, Flavor component accumulation

## Abstract

To interpret the formation characteristic flavor during oolong tea manufacturing process, the dynamic changes of key flavor components in samples from various processing steps of Tieguanyin oolong tea production were investigated using widely-targeted metabolomic and the transcriptomic approaches. As a result, a total of 1078 metabolites were determined, of which 62 compounds were identified as biomarkers significantly changed over the manufacturing process. Quantitative determination of the total 50,343 transcripts showed 7480 of them were co-expressed different genes. Glutamic acid served as a critical metabolism hub and a signaling molecule for diverse stress responses. Additionally, the targeted quantification results showed that the contents of catechins and xanthine alkaloids in dried tea were dramatically decreased by 20.19% and 7.15% respectively than those in fresh leaves, which potentially contributed to the alleviation of astringent or bitter palates, promoting the characteristic mellow and rich flavor of Tieguanyin oolong tea.

## Highlights

• Glutamic acid serves as a critical metabolism hub for diverse stress responses.
• Long-term setting is conducive to the accumulation of key flavoring compounds.
• Reduction in bitter/astringent components contributed to the mellow/rich flavor.

## 1 Introduction

Tieguanyin is a semi-oxidized oolong tea originated from Anxi county, Fujian province, China. Tieguanyin tea is popular in southern China and widely consumed in Southeast Asia due to its refreshing, brisk taste, mellow mouthfeel, and health benefits (Cao et al., 2022). Non-volatile compounds account for the taste flavor of oolong tea, which are formed and accumulated over the enzymatic-catalyzed process (ECP) and enzymatic-catalyzed process (NCP). The ECP is composed of the steps such as solar/indoor withering, turning-over/setting steps, while the NCP includes the steps such as firing, rolling, and drying steps during the manufacturing procedure of oolong tea production (Wu et al., 2020a). The tea shoots are kept alive and submitted to multi-stress such as solar radiation, water-loss, and mechanical wound during ECP stage, which induce dynamic changes of metabolites in response to adverse environment (Zeng et al., 2019), providing the material basis for the subsequent process of NCP and facilitating the final quality of oolong tea (Wu et al., 2022). Environmental stresses such as wounding, dehydration, and ultraviolet irradiation over ECP stage are important elicitors for Tieguanyin characteristic flavor, which is formed due to the stress-responsive biochemical reactions of tea tree (Zheng et al., 2022a). Additionally, dynamic changes of non-volatile compounds, such as amino acids, flavonoids, and alkaloids triggered by multiple stresses occurred over the manufacturing procedure, contribute to the specific flavor of oolong tea (Huang et al., 2020). Alkaloids and flavonoids exhibited extensive bioactivities, and were considered as the most effective components in oolong tea (Wang et al., 2019). However, limited attention has been paid to illustrating the flavor associated non-volatile metabolic changes over oolong tea production.

The integrated multi-omics analysis has been increasingly used to elucidate the biological process and quality control in tea production. Metabolomics approaches provide a comprehensive insight into the key compounds and relevant flavor in teas (Fan et al., 2021, Song et al., 2022). The integrated analyses combining transcriptomic and metabolomic datasets have been adopted in tea plant to obtain a further understanding on metabolic and molecular mechanisms underlying physiological processes in response to exogenous stimulus (Cheng et al., 2023).

In this study, freshly plucked tea shoots from *Camellia sinensis* cv. Tieguanyin were subjected to the sequential manufacturing processes. The aim of this study was to interpret the dynamic changes by using metabolomic and transcriptomic dataset, which would provide a deeper understanding of biochemical regulation on the metabolic pathways and the flavor development over the production process of Tieguanyin tea.

## 2 Materials and methods

### 2.1 Tea samples and reagents

The banjhi tea shoots composed of two or three leaves with an apical bud were plucked from *Camellia sinensis* cv. Tieguanyin cultivated on the tea garden of Laogu Tea Specialized Cooperative (Gande Town, Anxi County, Fujian Province, 25.17 °N, 117.51 °E) in April 2022. Then the collected tea shoots were subjected to the sequential production process according to the traditional procedure. In brief, the detached tea shoots were exposed to solar withering for 35 ∼ 45 min on mats, followed by indoor withering for approximate 20 min on bamboo sieves. Afterwards, the first turning-over was performed within 2 min in a rotary machine (90S, coupled with 1.5 m × 6 m turning-over bucket, Nandan machinery factory, Anxi County, China), followed by a setting step on bamboo sieves for 1 h; then the second turning-over was carried out within 4 min, followed by a 1 h setting; the third turning-over lasted for 5 min, with another setting indoor for 12 h prior to firing step. The tea shoots were heated in an oven for approximate 5 min at 260 ∼ 320 °C, and then the tea shoots were packed rolled at room temperature and dried at 110 °C until the moisture content declined to less than 6 %.

For metabolomic analysis, tea samples were collected at each step of ECP stage and from the dried tea, as presented in Table 1. For transcriptomic analysis, only the tea shoots in ECP stage were collected (Table 1), as the RNA was inactivated upon the firing treatment. The second leaf basipetal from the apical tip was carefully cut off with scissors and incubated in liquid nitrogen for 10 min. Then, the samples were placed in dry ice for transportation and stored at −78 °C in a refrigerator (DW84L326, Haier, China). Each of the tea sample consisted of three independent biological replicates. The moisture contents of tea samples collected in each step were measured using MB120 moisture-meter (OHAUS Company, NJ, USA).

**Table 1:** The moisture contents and appearance of tea leaves during manufacturing process of Tieguanyin oolong tea production.

Stage	Manufacturing process	Sample ID	Moisture content (%) a	Image	Detection method b
Enzymatic-catalyzed process	Fresh leaf (just pluck from Tieguanyin tea plant)	FL	72.84 ± 0.54 A		M + T
	After withering	WT	70.13 ± 1.06B		M + T
	After 1st turning-over	T1	67.35 ± 0.86C		M + T
	After 2nd turning-over	T2	63.22 ± 0.82 D		M + T
	After 3rd turning-over	T3	62.59 ± 1.06 D		M + T
	Before firing	BF	66.81 ± 0.35C		M + T
Non-enzymatic-catalyzed process	Dried tea	DT	3.59 ± 0.29 E		M

a The different capital letters represented significant difference (*P* < 0.01) of moisture contents among Tieguanyin tea samples.

b M: metabolomic determination; T: transcriptomic determination.

Acetonitrile and Methyl alcohol with chromatographical grade, were purchased from Merck Corporation (Darmstadt, Germany). The UPLC reference compounds were purchased from Sigma-Aldrich (Shanghai, China), all other reagents with analytical grade were obtained from Sinopharm Reagents Corporation (Shanghai, China).

### 2.2 The targeted and widely-targeted metabolomic analysis on non-volatile compounds in Tieguanyin tea samples

The targeted quantification on catechins, xanthine alkaloids and amino acids was determined using an ultra-performance liquid chromatography (UPLC) system. After the tea sample was thawed and smashed, an amount of 0.05 g of the sample was mixed with 500 µL of 70 % methanol. The tea sample was vortexed for 3 min under the condition of 2,500 r/min and centrifuged at 12,000 r/min for 12 min at 5 °C. Take 300 μL of supernatant into a new centrifuge tube and place the supernatant in –22 °C refrigerator for 25 min, then the supernatant was centrifuged at 12, 000 r/min for 15 min at 5 °C. After centrifugation, 200 μL of supernatant was used for further LC analysis. The UPLC system (ExionLC AD system, SCIEX Pte. Ltd., Singaproe) conditions were as follows: ACQUITY BEH Amide (i.d.2.1 × 100 mm, 1.7 μm); mobile phase: water with 2 mmol/L ammonium acetate and 0.04 % formic acid (A), acetonitrile with 2 mmol/L ammonium acetate and 0.04 % formic acid (B); The gradient was started at 90 % B (0–1.2 min), decreased to 60 % B (9 min), 40 % B (10–11 min), finally ramped back to 90 % B (11.01–15 min); flow rate, 0.4 mL/min; temperature, 39 °C; injection volume: 2 μL. For targeted quantification, peak areas were compared to an external standard solution.

The identification and quantification of widely-targeted metabolites were carried out by MetWare Company (Wuhan, China) based on the method reported previously (Wu et al., 2020a). In brief, the samples were freeze-dried and milled by a grinder (MM 500 VARIO, Retsch Company, Germany) for 150 s on 30 Hz. Subsequently, the powder (100 mg) was extracted using 1 mL 75 % methanol containing 0.1 mg L^-1^ lidocaine as the internal standard at 5 °C for 12 h. The mixture was subsequently centrifuged at 12,000 × g for 15 min at 5 °C, and the supernatant was collected and filtered using 0.22 µm filter (Millipore Corp., UK) before further detection. The obtained solution was subjected to metabolomic analysis on an UPLC electrospray ionization tandem mass spectrum system (Applied Biosystems 4500 QTRAP, Thermo Fisher Corporation, Waltham, USA). The ultra-performance liquid chromatography conditions were set according to the protocol used in previous work (Wu et al., 2020a). The electrospray ionization source operation parameters were as follows: source temperature 550 °C; ion spray voltage (IS) 5500 V (positive ion mode)/−4500 V (negative ion mode); ion source gas I, gas II, and curtain gas were set at 50, 60, and 25 psi, respectively; the collision-activated dissociation was high. Instrument tuning and mass calibration were performed with 10 and 100 μmol/L polypropylene glycol solutions in triple quadrupole (QQQ) and linear ion trap (LIT) modes, respectively. QQQ scans were acquired as electrospray ionization (MRM) experiments with collision gas (nitrogen) set to medium. DP (declustering potential) and CE (collision energy) for individual MRM transitions was done with further DP and CE optimization. A specific set of MRM transitions were monitored for each period according to the metabolites eluted within this period. The identification and quantification of each metabolite were conducted by comparing the retention time, mass-to-charge values, and the fragmentation patterns with authentic standards, or blasting against the internal and public databases (KNApSAcK, MassBank, METLIN, and MoTo DB). To evaluate reliability and repeatability of analytical results, a quality control (QC) sample by mixing 15 g of each sample powder in triplicate was prepared and subjected to determination.

### 2.3 RNA-sequencing analysis on Tieguanyin tea samples

The total RNA extraction of each sample was carried out using TRIzol reagent (Invitrogen, NJ, USA.) according to the manufacturer’s protocol. The RNA integrity was measured by 1 % agarose gel electrophoresis, and the RNA concentration was detected on Qubit2.0 Flurometer (Life Technologies, CA, USA). An aliquot of RNA per sample (1 μg) was subjected to RNA-sequencing analysis. Briefly, mRNA was purified from total RNA using poly-T oligo attached magnetic beads. Fragmentation was performed using divalent cations, and first-strand cDNA was synthesized using random hexamer primer and reverse transcriptase. Subsequently the second strand cDNA synthesis was carried out using DNA Polymerase I and RNase H. The obtained cDNA was purified with AMPure XP system (Beckman Coulter, Beverly, USA). The size-selected, adaptor-ligated cDNA was then treated with 3 μL USER Enzyme (NEB, USA) at 37 °C for 20 min, followed by incubation at 95 °C for 6 min before PCR. The PCR was performed using Phusion High-Fidelity DNA polymerase, Universal PCR primers and Index (X) Primer, and the PCR products were purified with AMPure XP system, then the library quality was evaluated at the Agilent Bioanalyzer 2100 system.

The raw reads filtration was carried out to obtain clean reads by removing adaptor and ambiguous sequences. The clear reads were blasted against the reference genome of *Camellia sinensis* cv. Tieguanyin (Zhang et al., 2021) using HISAT v2.1.0. The quantification of gene expression levels (Fragments Per Kilobase of exon model per Million mapped fragments, FPKM) were performed using featureCounts v1.6.4 and StringTie v1.3.4. The analysis on differential expressed mRNAs in paired-comparisons was conducted on DESeq2. The transcripts with false discovery rate (FDR) parameter less than 0.05 and |log_2_foldchange|≥1 were considered as different expressed genes (DEGs). Enriched metabolic pathways and gene functions were annotated based on Kyoto Encyclopedia of Genes and Genomes (KEGG) database.

### 2.4 The electronic tongue (E-tongue) analysis

The taste features of Tieguanyin samples were evaluated using E-tongue system (TS-5000Z, Insent Electricity Company, Japan). In brief, the powder of each sample (3.0 g) was infused with 150 mL freshly boiled water for 5 min, then the tea brewing was quickly filtrated and cooled to 25 ℃ before taste attribute assessment. E-tongue system was composed of a reference probe and a panel of sensor probes, the sensor probes are dipped into the tea brewing and reference solution to detect the taste intensity. The sensor probes include astringency, bitterness, aftertaste-astringency, aftertaste-bitterness, sourness, richness, umami, sweetness, and saltiness. A standard solution prepared by dissolving 30 mmol/L potassium chloride and 0.3 mmol/L tartaric acid in distilled water was served as cleaning and reference solution. The thresholds for saltiness and sourness and were −6 and −13, respectively, and zero for other tastes. The obtained taste scores were applied to further analysis to depict the taste features of the samples of Tieguanyin tea. Each tea sample was composed of three independent biological replicates.

### 2.5 Data analysis

The analysis on the different metabolites in paired-comparisons and the visualization of the metabolomic dataset were carried out by using principal component analysis (PCA), hierarchical cluster analysis (HCA), and orthogonal projection to latent structures discriminant analysis (OPLS-DA) by using R. The criteria with log_2_|(Fold change)| higher than 1, and variable importance projection (VIP) values more than 1.0 for each comparison was applied to predict the significant different metabolites in this study.

## 3 Results and discussion

### 3.1 The dynamic changes of phenotypic profiles of Tieguanyin samples during production process

Table 1 displays the moisture contents of the Tieguanyin tea sample throughout the production process. Dehydration during the ECP stage resulted in the decreasing of water contents of Tieguanyin tea leaves from 72.84 % (FL) to 62.59 % (T3), then the moisture content in BF sample fluctuated at 66.81 % (Table 1), indicating that moisture from the tea stems was transferred to the leaves during the 12-hour setting before firing treatment. The similar result is also observed in the previous research that water content was increasing after the long term setting step of oolong tea production (Zeng et al., 2022). The appearance of Tieguanyin tea shoots over the ECP stage became gradually wilted, and the color turned into dark-green, with the gloss fading away (Table 1), indicating dehydration of the leaf cells. This decreases the mechanical performance against external forces and leads to leaf cell destruction over the turning-over step (Liu et al., 2022). This facilitates immediate contact of the intra-vacuole compounds and the enzymes stored in cytoplasm, which in turn contributes to the enzymic-catalyzed reactions and development of tea flavor.

### 3.2 The dynamic changes of non-volatile components over Tieguanyin tea production

**Fig. 1:**
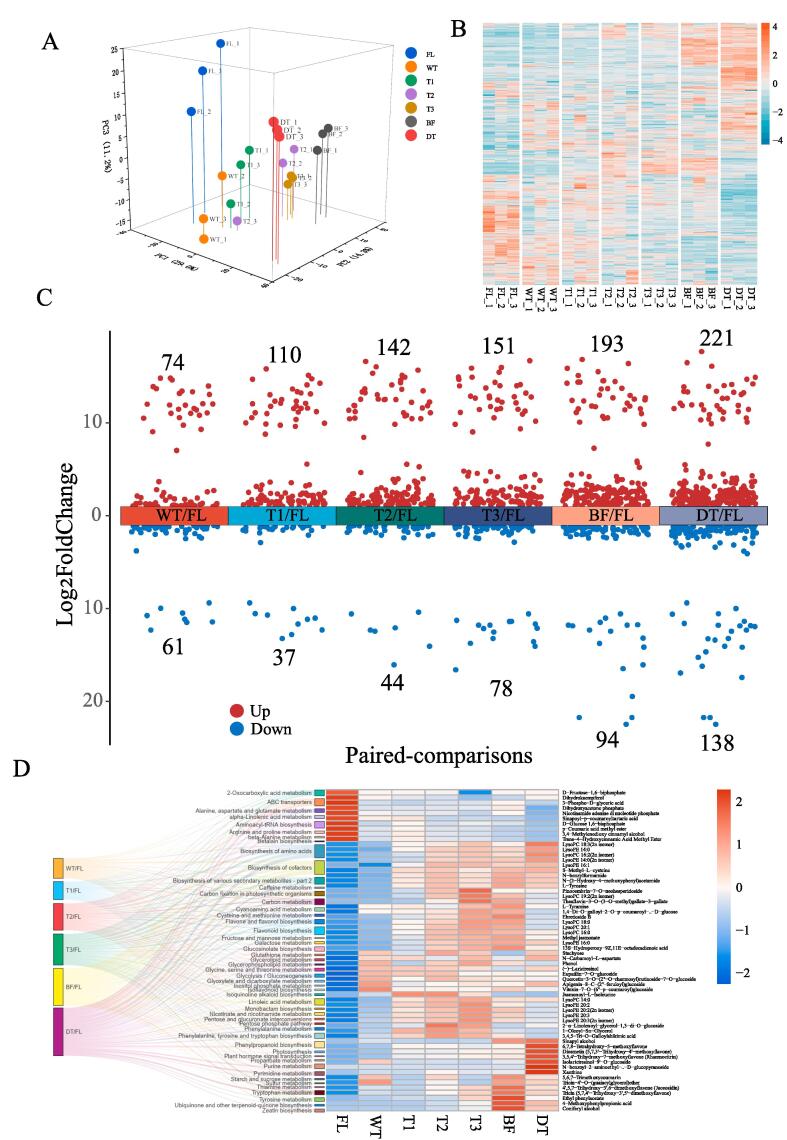
The profiles of non-volatile metabolites from Tieguanyin oolong tea samples during different producing process. (A) The PCA score plot of different leave samples based on metabolomic dataset. (B) The heatmap of all the identified metabolites from Tieguanyin oolong tea samples. (C) The number of significant differential metabolites in various paired-comparisons with the criteria of Log_2_ |(Fold change)| ≥ 1 and VIP values ≥ 1.0. The numbers labeled on each comparison represented the amounts of up- or down-regulated metabolites. (D) The pathway clustering analysis based on the paired-comparisons and the significant different metabolite profiles obtained from the paired-comparisons. FL: fresh leaf; WT: after withering; T1: after 1st turning-over; T2: after 2nd turning-over; T3: after 3rd turning-over; BF: before firing; DT: dried tea.

A widely-targeted metabolomic determination approach was used in this study to identify and quantify non-volatile components over Tieguanyin tea production. As a result, a total of 1078 non-volatile components were identified, mainly including 285 flavonoids, 96 lipids, 88 amino acids derivatives, 72 alkaloids, 70 organic acids, 65 nucleotides derivatives (Table S1). The multi-peak detection graph and total ions current of quality control graph (Fig. S1) demonstrated that the metabolomic data detected in this work have a good reliability and repeatability. The three-dimensional PCA result of metabolomic profiles of different Tieguanyin tea samples showed that PC1, PC2 and PC3 accounted for nearly 60 % of total variance (Fig. 1A). The score plot of PCA showed each sample could be clearly distinguished from others, while the triplicates of the same sample were closely clustered (Fig. 1A), suggesting a dynamic change of metabolites accumulated in tea leaves from different steps occurred over Tieguanyin tea production process. The heatmap of quantified metabolites from different samples showed that the abundance of metabolites was dramatically altered over the production process of Tieguanyin tea (Fig. 1B), which is agreed with the result of PCA. These dynamic changes of metabolites were predominantly evoked by the multiple-stress elicited enzymatic-catalyzed reactions over ECP stage, subsequently consolidated by thermal effect that occurred during firing and drying steps of oolong tea manufacturing, which are congruous with the previous report (Liu et al., 2022).

To further investigate the impact of each manufacturing step on the non-volatile components of Tieguanyin tea, the significant different regulated non-volatiles (DRNs) based on the OPLS-DA of processed leaf vs. fresh leaf (FL) were analyzed with the criterial of VIP ≥ 1 and Log_2_| Fold change | ≥ 1 (Fig. 1C, Table S2). As showed in Fig. 1C, the numbers of DRNs were increasing with the process of Tieguanyin tea manufacturing, with the most abundant DRNs observed in DT/FL (221 up-regulated, 138 down-regulated). Only 135 DRNs (74 up-regulated, 61 down-regulated) were observed at the early stage of ECP (WT/FL), suggesting the enzymatic-catalyzed conversion of non-volatile compounds is a cumulative process, and then the thermal effect in firing and drying process promoted the alterations of the non-volatiles.

Furthermore, the shared DRNs and co-expressed pathways involved in all the paired-comparisons were screened out to explore the dynamic changes in metabolites throughout the Tieguanyin oolong tea manufacturing process. A total of 50 pathways were enriched in the co-expressed pathways analysis, including “biosynthesis of amino acids”, “flavonoid biosynthesis”, “glutathione metabolism” *etc*. (Fig. 1D), which are also observed during the oolong tea production in our previous study (Wu et al., 2020a). Moreover, a total of 62 metabolites were dramatically regulated over the entire production process (Fig. 1D). Notably, the metabolites in glycolysis such like d-glucose 1,6-bisphosphate, d-fructose-1,6-biphosphate, 3-phospho-d-glyceric acid, and dihydroxyacetone phosphate displayed a decreasing trend along Tieguanyin tea production. These carbohydrate derivatives potentially function as precursors or signaling molecules over metabolic processes in response to adverse environment conditions, such as water deficit, osmotic stress or extreme temperature in plant (Thalmann & Santelia, 2017).

As the major components of biological membranes, lysophospholipids serve as the signaling molecules associated with various adverse environmental responses, and they are produced substantially under adverse conditions such as drought and extremely temperature (Okazaki & Saito, 2014). In this study, the contents of fatty acid derivatives, including lysophosphatidyl cholines (LysoPCs) and lysophosphatidyl ethanolamines (LysoPEs) increased along with ECP stage of Tieguanyin tea manufacturing (Fig. 1D), as fatty acid derivatives are potentially involved in the regulation of stress signaling in the leaves and contribute to the development of flavor over ECP stage of oolong tea production (Guo et al., 2022). Methyl jasmonate (MeJA) and jasmonoyl-isoleucine (JA-Ile), derived from fatty acids metabolism, also increased during withering and turning-over steps (Fig. 1D). This result is consistent with the previous study that jasmonic acid (JA) derivatives were massively produced as elicitors that triggered the downstream biological process in response to the water loss and mechanical wound during ECP stage (Wu et al., 2022).

### 3.3 The analysis on the transcriptomic profiles over ECP stage of Tieguanyin tea production

**Fig. 2:**
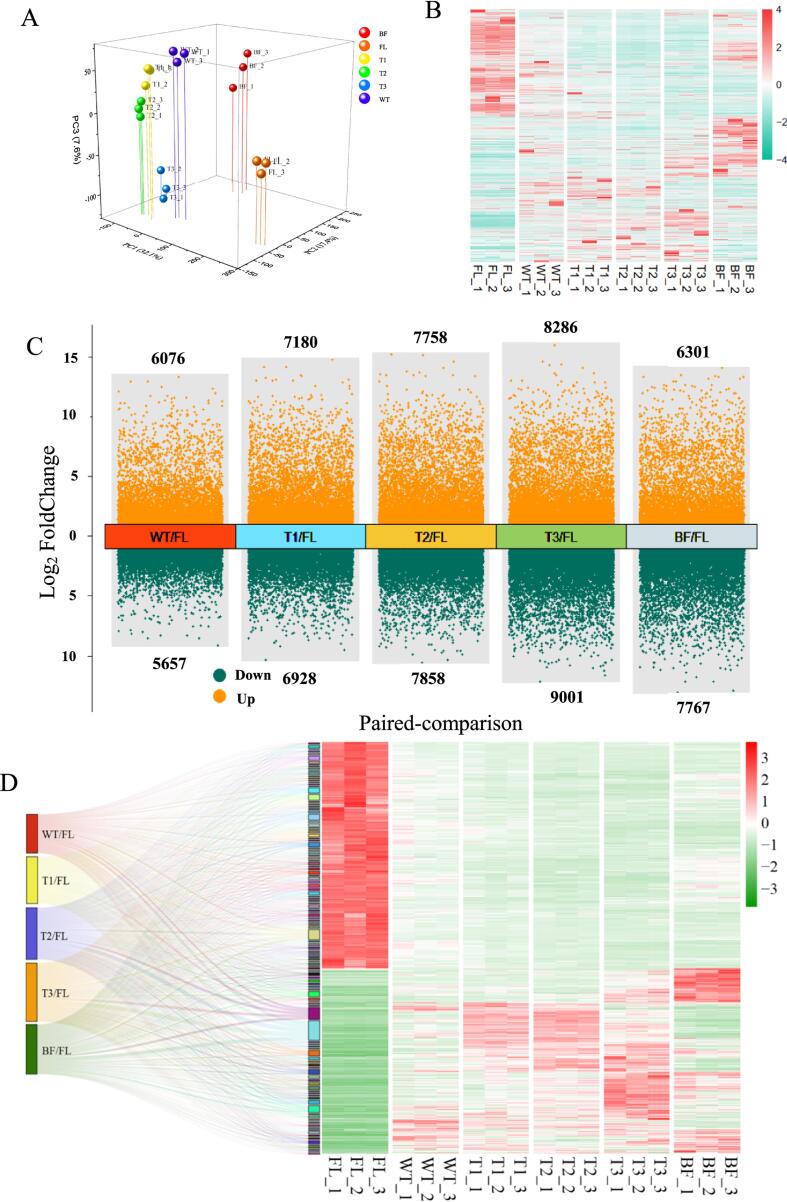
The profiles of RNA-seq dataset from Tieguanyin oolong tea samples during different producing process. (A) The PCA score plot of different leave samples based on transcriptomic dataset. (B) The heatmap of all the identified genes from Tieguanyin oolong tea samples. (C) The number of significant differential genes in various paired-comparisons with the criteria of Log_2_ |(Fold change)| ≥ 1 and false discovery rate < 0.05. The numbers labeled on each comparison represented the amounts of up- or down-regulated genes. (D) The pathway clustering analysis based on the paired-comparisons and the significant different gene profiles obtained from the paired-comparisons. FL: fresh leaf; WT: after withering; T1: after 1st turning-over; T2: after 2nd turning-over; T3: after 3rd turning-over; BF: before firing.

A total of 50,343 transcripts were identified through RNA-sequencing in this study, of which 15,367 were novel genes. The PCA score plot demonstrated that the transcriptomic profiles of different samples were well-separated from each other, while the triplicates of each sample were comparatively clustered, with the total variance of first three PCs accounting for 57.1 % (Fig. 2A), showing a fine representativeness of the samples. The expression of transcriptomic profiles was presented in Fig. 2B, in which the heatmap displayed the dynamic trend of the transcripts, and a chronological model of stress-induced gene alternation was observed. This result is congruous with the previous study on the dynamics of wounding-induced gene regulation during oolong tea production (Zheng et al., 2022a). The number of DEGs in paired-comparisons of tea samples were presented in Fig. 2C. Generally, the number of DEGs showed an increasing tendency during ECP stage, with maximum DEGs observed in T3/FL (8,286 up-regulated and 9,001 down-regulated), which is similar with the trend of DRNs (Fig. 1C). This result suggests that transcriptional burst event occurred in tea leaves as a conservative defense strategy in response to the external stress, such as mechanical wounding and water loss. Additionally, the burst on transcript level gradually recovered over the setting prior to firing treatment, contributing to the reduction of DEGs in BF/FL (6301 up-regulated, 7761 down-regulated, Fig. 2C), as the defense response in tea leaves induced by mechanical wounding faded away during the 12-hour setting.

To further explore the crucial pathways associated with the physiological processes in response to adverse conditions during Tieguanyin oolong tea production, DEGs shared in all paired-comparisons were screened. A total of 7,480 co-expressed DEGs were identified, clustered into 136 pathways, including “starch and sucrose metabolism”, “plant-pathogen interaction” and “mitogen-activated protein kinase (MAPK) signaling pathway - plant” (Fig. 2D). Plant defense system would be altered under multifactorial stress, especially the elevated temperatures have been implicated in interfering with metabolic and transcriptomic modifications relative to plant-pathogen interaction (Prasch & Sonnewald, 2013). During the stress-response process, sugar metabolism and mobilization are greatly affected, the allocation of sugar is modulated to counterbalance the negative impacts of abiotic stresses (Saddhe et al., 2021). The multifactorial stress can disrupt various signaling defense pathways, such as MAPK cascades, which are highly conserved signaling transduce extracellular stimuli into intracellular responses (Meng & Zhang, 2013). The dynamic changes of the co-expressed DEGs could be roughly divided into two trends: (i) decreasing as the production process proceed; (ii) increasing as the production process proceed (Fig. 2D). This result is congruous with the observation in the previous study that the differential gene expression induced by stress were alternatively up- or down-regulated during oolong tea post-harvest (Zheng et al., 2022b).

### 3.4 The transcriptome-metabolite integrated analysis on flavor constitutes accumulation

**Fig. 3:**
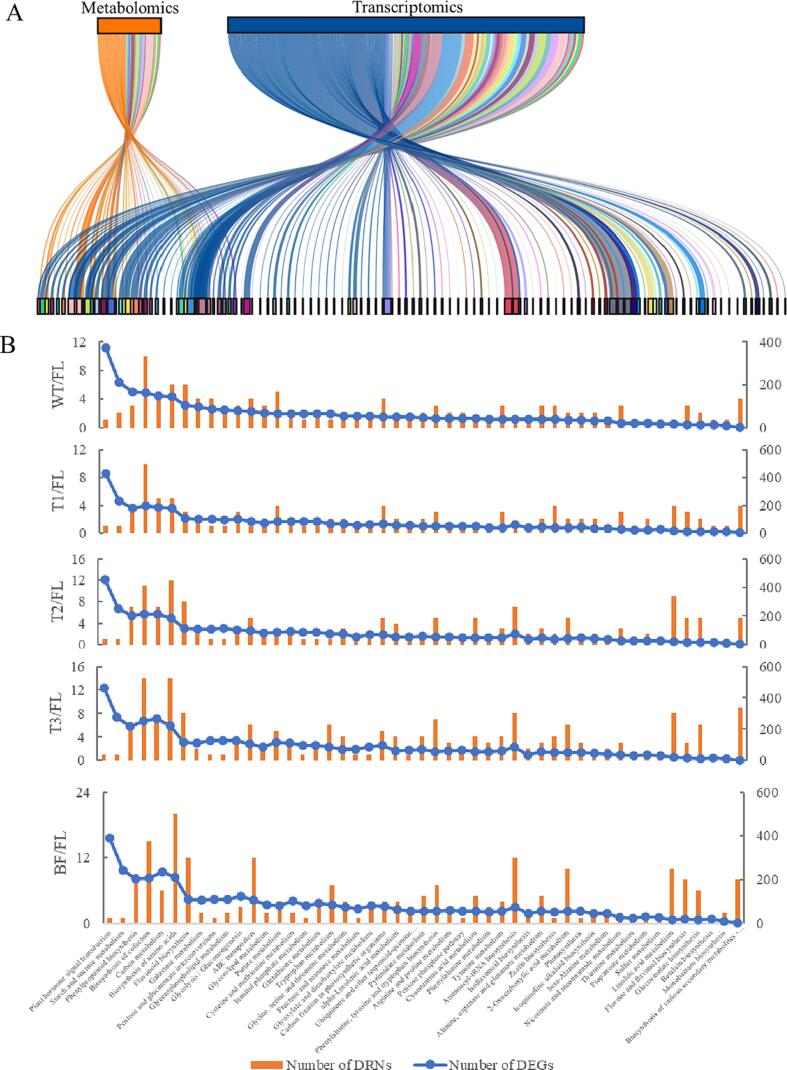

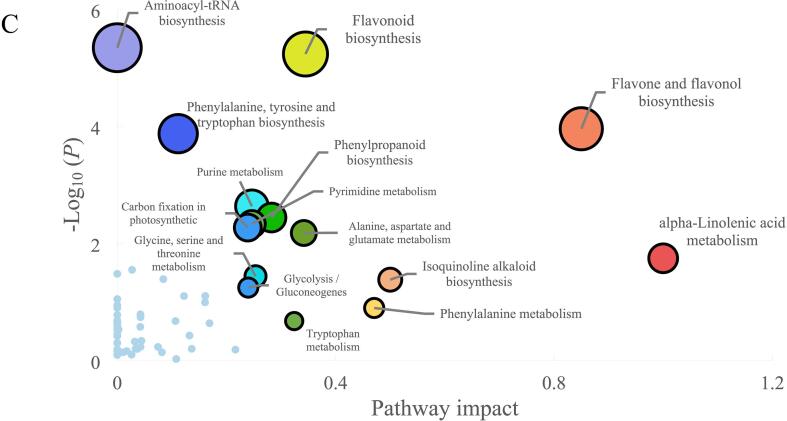
The co-expressed pathways identified from the metabolomic and transcriptomic profiles of Tieguanyin tea samples of ECP stage. The identified pathways from the metabolomic and transcriptomic profiles (A). The number of DRNs and DEGs associated with the co-expressed pathways identified in the paired-comparisons of WT/FL, T1/FL, T2/FL, T3/FL, and BF/FL (B). The integrative pathway analysis (C). FL: fresh leaf; WT: after withering; T1: after 1st turning-over; T2: after 2nd turning-over; T3: after 3rd turning-over; BF: before firing.

A complementary analysis of metabolomic and transcriptomic datasets was performed applied to explore the co-expressed functional pathways during the enzymatic-catalyzed biological processes over ECP stage of Tieguanyin oolong tea production. Totally, 49 co-expressed pathways were obtained, mainly including “α-Linolenic acid metabolism”, and “Biosynthesis of amino acids” (Fig. 3A). Furthermore, the DRNs and DEGs involved in the paired-comparisons were filtered out to investigate the relationships between molecular biological processes and metabolite accumulation. As showed in Fig. 3B, the amounts of DRNs were generally consistent with DEGs in each clustered pathways, suggesting various genes altered during each step of oolong tea processing, resulting in the changes in metabolite accumulation. The number of DRNs and DEGs showed an increasing tendency during the turning-over stage, peaked at the T3 steps (Fig. 3B), suggesting the continuous environmental stress such as mechanical wounding, drought and low temperature, stimulated leaf cells, subsequently the intracellular cascades were initiated to orchestrates the response to abiotic stress and drive alterations in metabolic networks for survival (Zeng et al., 2019). The number of DEGs in tea leaves decreased after a 12-hour setting before BF step, as part of later stage of ECP process. However, the amount of DRNs at BF step remained at a level consistent with previous steps (Fig. 3B). This inconsistency between transcriptomic and metabolomic profiles was also observed in the study on the development of young shoots of tea plants, which was ascribed to the hysteretic nature among the chronological sequence of transcription, translation, enzymatic-catalysis and metabolomic accumulation (Wu et al., 2020b).

The “Plant hormone signal transduction” pathway exhibited a contrast between the amounts of DRNs and DEGs. Specifically, only the jasmonoyl-l-isoleucine (JA-Ile) was predicted as a DRN in this pathway over the paired-comparisons (Fig. 3B). JA derivatives (JAs) play critical roles in responses to abiotic stress, including drought and heat. Under such adverse conditions, JA-Ile is formed in cytosol and transported to nucleus, where it interplayed with F-box protein CORONATINE INSENSITIVE1 (COI1) and JASMONATE-ZIM-DOMAIN (JAZ), resulting in release of transcription factors to modulate the expression of JA-responsive genes. (Devireddy et al., 2021). The higher concentration of JA-Ile during the ECP stage than in fresh leaves suggests that adverse stimuli rapidly altered the level of JAs, continuously eliciting the JA signaling pathway to manipulate the molecular processes as a resistance mechanism when exposed to abiotic stress. Similarly, glucose 1,6-bisphosphate was identified as DRN in “Starch and sucrose metabolism” pathway over paired-comparisons (Fig. 3B). Stress-induced starch hydrolysis increases the concentration of glucose derivatives, acting as a sugar-source when rapid energy supply or intracellular signals are required for protective functions (Dong & Beckles, 2019). The dynamic changes of glucose derivatives in starch and sucrose metabolism pathway indicated that catabolism of carbohydrates in tea leaves occurred to buffer against the adverse effects of stress, ensuring the short-term survival of leaves to facilitate the accumulation of flavoring compounds during oolong tea production.

To uncover the key pathways involved in the biological process in response to the adverse conditions over Tieguanyin tea production, a joint pathway analysis was conducted by using the metabolomic and transcriptomic profiles on online platform MetaboAnalyst (https://www.metaboanalyst.ca/, accessed on 25 April 2023). As presented in Fig. 3C, a total of 59 pathways were identified, with top 20 enriched pathways primarily involved in amino acid metabolism, glycolysis, α-linolenic acid metabolism, flavonoid biosynthesis, flavone and flavonol biosynthesis. This result suggests that environmental stimuli occurred during the ECP stage predominantly regulated the genes and compounds involved in these metabolic processes, potentially leading to the formation or modulation of characteristic flavor of Tieguanyin tea. Certain abiotic stresses trigger phospholipases to release α-linolenic acid from membrane lipids, α-linolenic acid metabolism derived JA-Ile is known to accumulate in plants in response to abiotic stresses, resulting in the α-linolenic acid pathway enriched in joint analysis (Fig. 3C). Additionally, abiotic stresses shift the focus of the plant metabolomic network from growth or development to stress resistance and damage tolerance through dramatical fluxes of the free amino acids, then the amino acids derived secondary metabolites accumulated under diverse stressful environmental conditions (Heinemann & Hildebrandt, 2021). The phenylalanine metabolism derived flavonoids, including catechins, flavones, chalcones, flavonols, *etc*., participate in plant defense process in response to a diverse set of stresses, such as extremely temperature, UV irradiation, as well as drought (Shomali et al., 2022). Therefore, most of the key pathways predicted in the joint analysis are clustered into amino acids metabolism or flavonoids biosynthesis clades as presented in Fig. 3C, coincidentally associated with the flavoring compounds metabolism during Tieguanyin oolong tea production.

### 3.5 The metabolic network of flavoring components constructing by joint pathways analysis

**Fig. 4:**
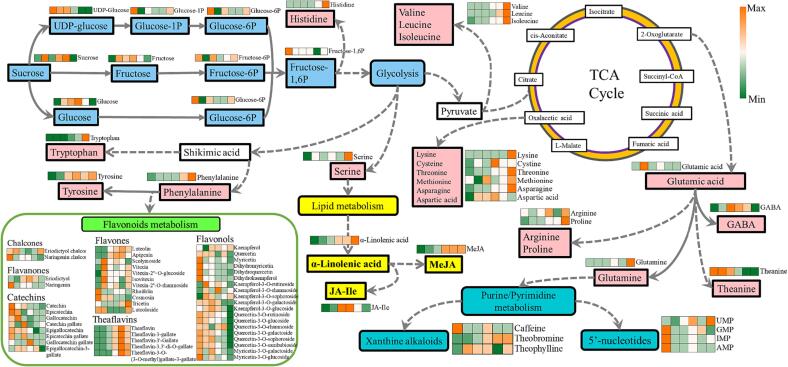
The predicted network diagram of metabolic pathways associated with flavor formation during ECP stage of Tieguanyin oolong tea production. The heatmap was plotted using log_2_-transformed values from the metabolomic dataset, the cells in the heatmap from left to right represented FL (fresh leaves), WT (withered leaves), T1 (leaves after 1st turning-over), T2 (leaves after 2nd turning-over), T3 (leaves after 3rd turning-over), and BF (leaves before firing) samples, respectively. TCA cycle: tricarboxylic acid cycle. UDP-glucose: Uridine diphosphate glucose. Glucose-1P: Glucose 1-phosphate. Glucose-6P: Glucose 6-phosphate. Fructose-6P: Fructose 6-phosphate. Fructose-1,6P: Fructose 1,6-bisphosphate. GABA: γ-Aminobutyric acid. GMP: guanosine 5′-phosphate; AMP: adenosine 5′-monophosphate; IMP: inosine 5′-monophosphate; UMP: uridine 5′-monophosphate. JA-Ile: Jasmonoyl-l-Isoleucine. MeJA: Methyl jasmonate.

To shed light on the alterations in metabolites and characteristic taste during ECP stage of Tieguanyin tea manufacturing, the metabolic network of flavoring components was constructed through the joint pathway analysis of the metabolomic and transcriptomic profiles (Fig. 4). Adverse conditions including drought, low temperature, oxygen stress could trigger the accumulation of soluble sugars such as sucrose, fructose, and glucose, which may function as osmoprotectants or reactive oxygen species (ROS) scavengers to mitigate negative effect of the stress (Saddhe et al., 2021). This finding is consistent with the result that the contents of sugars began to accumulate under the multiple stresses at withering step, peaked during the turning-over step of Tieguanyin tea production (Fig. 4). Apart from directly participating in stress resistance, sucrose and its hydrolytic product act as the signaling compounds that could be sensed and further relayed via glycolysis to launch the accrual of anti-stressor molecules (Salvi et al., 2022), probably leading to the reduction in the contents of sugar derivatives during the 12 h setting before firing step as presented in Fig. 4.

The maximum contents of most amino acids were reached at BF step as shown in Fig. 4. This finding is agreed with the previous observation amino acids surged in the fermentation process of tea production because of the enzymatic hydrolysis of low-molecular-weight proteins or peptides (Deb & Pou, 2016). As a potential signaling function in response to abiotic stress such as UV irradiation and drought, shikimic acid derived phenylalanine (Phe) or tyrosine (Tyr) would massively accumulate in plants; similarly, pyruvate derived aspartic acid (Asp) and asparagine (Asn) are involved in plant stress resistance in response to drought, cold or alkaline salt (Heinemann & Hildebrandt, 2021). The levels of Phe, Tyr, Asp, and Asn showed gradually increasing trend along with ECP stage of Tieguanyin tea production (Fig. 4), which may be the result of the utilization of these amino acids as signaling components or alternative substrates in dealing with diverse stressful conditions in tea leaves.

Glutamic acid (Glu) is a signaling molecule associated with response or acclimation to environmental stress, such as heat, cold, drought, and wound. As demonstrated in Fig. 4, the content of Glu in the tea leaves peaked at WT step, probably attributing to the induction of heat or drought stress encountered during withering process; afterward the reduction of Glu over the entire turning-over step was observed (Fig. 4), which could be ascribed to its metabolism in plants. This result is agreed with the observation that Glu reached the maximum content during withering step and significantly reduced during turning-over stage of Zhangping Shuixian oolong tea production (Wu et al., 2022). As a universal precursor of several flavoring components, Glu takes part in the biosynthesis of proteinaceous amino acids (proline, glutamine, arginine) and non-proteinaceous amino acids (γ-aminobutyric acid, l-theanine). Proline (Pro) acts as a major osmoprotectant in plants, and arginine (Arg) serves as a regulator on defense mechanism against stress by fine-tuning the biosynthesis proline and polyamines (Hildebrandt, 2018). The dramatical accumulation of Pro and Arg at the end of ECP (Fig. 4) indicates these amino acids may account for balancing the increased vacuolar osmolarity in response to the long-term dehydration during ECP stage of Tieguanyin tea production. Glu derived γ-aminobutyric acid (GABA), is massively generated in plants in response to environmental stimuli. The abundance of GABA in the tea samples showed a fluctuant trend with the plateau values observed in turning-over stage (Fig. 4), suggesting that the accumulation of GABA in Tieguanyin tea leaves was responding to abiotic stress, as the increased level of GABA was able to inhibit the stress-triggered excessive ROS production by activating antioxidant enzymes (Seifikalhor et al., 2019). l-theanine (Thea) accounts for over 50 % of total free amino acid in the greatest amount in tea leaves; however, as a non-proteinaceous amino acid, Thea is primarily biosynthesized in root, and transported via vascular tissues to the new shoots (Lin et al., 2023). The Thea level would be gradually decreased as the progress in manufacturing process because the content of Thea cannot be replenished by the hydrolysis of proteins, leading to the continuous decline of Thea during Tieguanyin tea production (Fig. 4).

In addition to participating in tolerance of drought and heat, Glu is also a hub connecting to downstream purine or pyrimidine metabolism, potentially resulting in the fluctuations in xanthine alkaloids and 5′-nucleotides (Fig. 4). The xanthine alkaloids, mainly including caffeine, theobromine, and theophylline, showed dynamic variations along ECP stage of Tieguanyin tea production. The level of caffeine was generally lowered, while the levels of theobromine and theophylline were enhanced from withering step (Fig. 4). This result is congruous with the previous observation that caffeine content in tea leaves decreased slightly at 3 h after a single mechanical injury and then increased continuously from 6 to 24 h (Li et al., 2018), while the continuous mechanical wound during the turning-over step contributed to the caffeine reduction, resulting in the accumulation of theobromine and theophylline via inter-conversion (Deng et al., 2020). The 5′-nucleotides such as guanosine 5′-phosphate (GMP), adenosine 5′-monophosphate (AMP), inosine 5′-monophosphate (IMP), and uridine 5′-monophosphate (UMP), influence the taste of umami as flavor enhancers. The contents of GMP, IMP as well as AMP were decreasing over ECP stage, in agreement with the observation that AMP and GMP gradually reduced over the withering process of tea production (Zhao et al., 2018).

The continuous dehydration and mechanical-wound could induce the elevation of Glu in plants, further activating glutamic acid receptors (GLRs) mediated Ca^2+^ signaling pathway. The cytosolic Ca^2+^ flux provokes JA synthesis, expanding its impact on other signaling pathways and regulatory processes (Farmer et al., 2020). The abundance of JA derivatives, including JA, JA-Ile and MeJA, showed a stepwise increase over ECP stage in this study (Fig. 4), suggesting that the effect of JA derivatives on regulation was strengthened as Tieguanyin oolong tea production proceeded, and the floral aroma originated from MeJA was facilitated the characteristic fragrance of Tieguanyin tea. However, further explore is needed for further understanding on regulation mechanism of JA on the metabolism of other secondary metabolites during ECP stage of oolong tea manufacture. As a precursor for biosynthesis of signaling molecules or secondary metabolites, Glu was closely associated with the formation of distinctive flavor of Tieguanyin oolong tea in this study, implying a crosstalk between Glu level and oolong tea flavor. Nevertheless, the interplay of Glu signaling with other signaling over ECP stage of oolong tea manufacturing also needs to be further solved.

Flavonoids derived from phenylalanine metabolism are crucial polyphenolic secondary metabolites associated with various processes of plant growth such as signaling, stress resistance and pigmentation. In this study, most of catechins, flavones, chalcones, flavanones, and flavonol glycosides accumulated in withering step (Fig. 4), due to the antioxidative effect of flavonoids in scavenging the superfluous ROS induced by UV exposure (Wu et al., 2023). Then, the contents of catechins and flavonol glycosides showed a generally decreasing trend over the turning-over steps (Fig. 4), in agreement with our previous study on the Zhangping Shuixian oolong tea production (Wu et al., 2022). The decrease in the levels of catechins and flavonol glycosides could be attributed to the down-regulated expression of genes associated with the flavonoid biosynthesis pathway as presented in Fig. S2. Moreover, the expression of peroxidase (POD) was enhanced to restrain intracellular oxidative stress caused by the multiple stress occurred during turning-over stage (Huang et al., 2020), catalyzing the conversion from catechins into theaflavin derivative (Fig. 4, Fig. S2). The contents of flavonol glycosides were clearly lowered at the final stage of ECP, as most of flavonol glycosides were degraded into glycoside moieties and aglycones, resulting in the alleviation of bitter or astringent intensity of teas (Fang et al., 2019).

The multi-stress such as continuous dehydration, mechanical wounding and low temperature took place during the withering and turning-over steps collectively induced the excessive production of ROS. The damaging and the signaling activities of ROS would lead to different impact on the level of gene expression profile and dynamic changes in molecules, in order to remove the intracellularly oxidative stress. An increase in the abundance of free amino acids under oxidative stress can result from both the activation of biosynthetic pathways and enhanced protein degradation over the production of several teas (Yu & Yang, 2020). These increased amino acids are used as resistant osmolytes, precursors for secondary metabolites, or storage forms of organic nitrogen. The phenylalanine derived secondary metabolites, such as flavonoids, are effective antioxidants and free radical scavengers, accumulation of these compounds is one of the most effective strategies to minimize oxidative damage. The continuous mechanical wounding occurred within the turning-over stage of Tieguanyin oolong tea production could be recognized by damage-associated molecular patterns on cell wall and then trigger ROS burst, resulting in the massive synthetization of stress hormone such as JA. The enhanced JA content further modulate the generation of flavonoids in response to the superfluous ROS (Sohn et al., 2022), which is in line with the short-term increase of flavonoids at the early stage of ECP in this study (Fig. 4). On the other hand, the gene expression and the enzymatic activities of POD and polyphenol oxidase (PPO) in tea plant increased as a response to the oxidative stress, which partially contributed to the oxidization of catechins in cell structure injured tea leaves, resulting in the decrease of catechins and increase in TFs observed at the later stage of turning-over in this study (Fig. 4). This suggests that sophisticated biochemical conversions occurred within the 12-hour setting after the turning-over step, and the long-term setting is indispensable and conducive to the quality of Tieguanyin tea.

Overall, the shifts on the amino acid and flavonoids metabolism in this study consolidates the view that the changes in biochemical process on transcriptomic and metabolic levels are aim to the prevention of ROS accumulation and production of antioxidative compound. The ECP stage of Tieguanyin oolong tea production takes advantage of the oxidative-stress response mechanism of tea leaves, to regulate the proportions of characteristic flavoring components such as free amino acids and flavonoids, facilitating the final quality of tea.

### 3.6 The impact of dynamic changes of metabolites on flavor of Tieguanyin tea

**Fig. 5:**
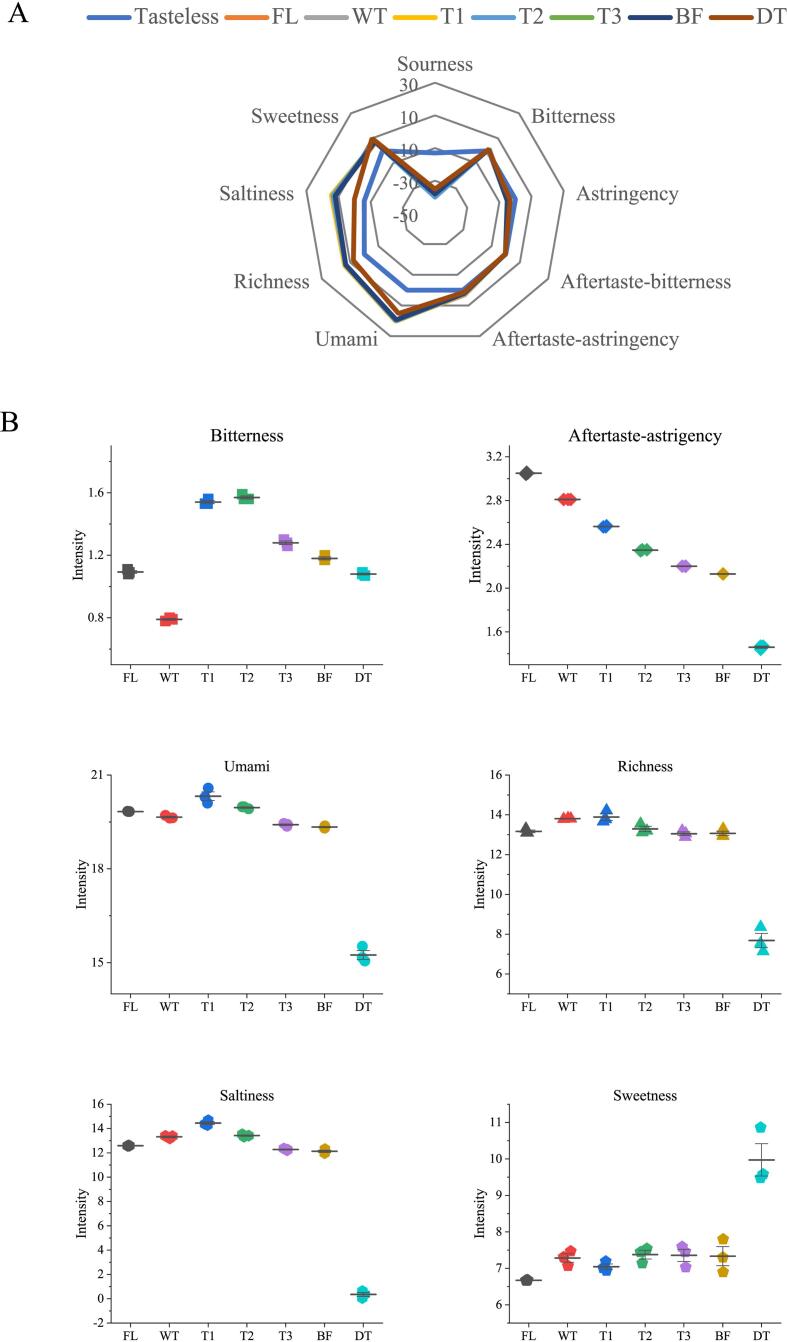

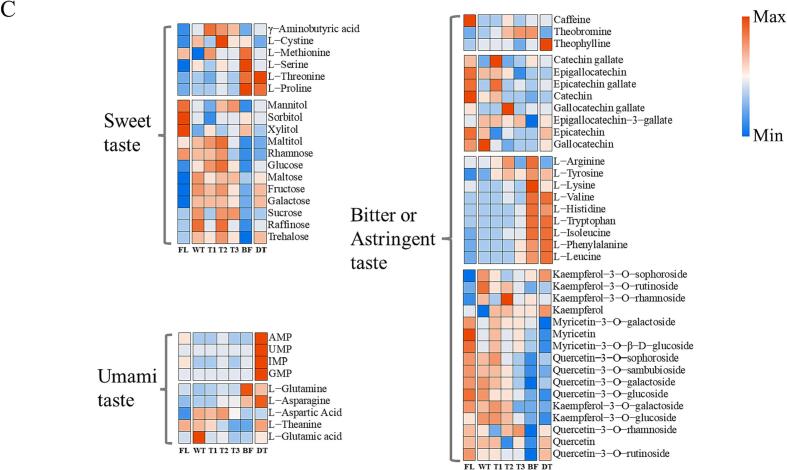
The plots of E-tongue dataset and heatmap of flavoring compounds in the samples from various steps of Tieguanyin tea production. The radar plot of E-nose dataset of different tea samples (A). The scatter interval plots of various palate attributes of different tea samples (B). The heatmap of dynamic changes of major flavoring compound over the manufacture procedure of Tieguanyin oolong tea production (C). The panel was based on the values of log_2_-normalized expression of metabolomics dataset. FL: fresh leaf; WT: after withering; T1: after 1st turning-over; T2: after 2nd turning-over; T3: after 3rd turning-over; BF: before firing; DT: dried tea.

E-tongue determination (Fig. 5 A & B) was performed to explore the impact of dynamic changes of metabolites on flavor of Tieguanyin tea in this study. The result showed that the detected sourness, astringency, and aftertaste-bitterness data were below the tasteless threshold (Fig. 5A), and thus the profiles of these tastes were discarded in this study. The intensities of aftertaste-astringency, bitterness, umami, saltiness, and richness in all tea samples showed decreasing variations with the process of Tieguanyin tea production (Fig. 5B). The intensity of bitter was decreased in withering step, which is congruous with the previous observation that bitterness taste of solar-withered tea leaves was significantly reduced due to the reduction of bitter taste compounds in withered leaves (Wang et al., 2022). Subsequently, the bitter intensity sharply enhanced and peaked at T3 step, followed by a gradual reduction until drying step (Fig. 5B), indicating that the accumulation of bitter taste compounds was decreasing at late stage of Tieguanyin tea production. The intensities of aftertaste-astringency, umami, saltiness, and richness showed slow and subtle changes over ECP stage, then dramatically dropped to the minimum values at drying step (Fig. 5B), suggesting that the sharp decline in these tastes in raw tea was ascribed to the thermal effect at drying step. Conversely, the intensity of sweetness in raw tea was markedly boosted after drying, which is in line with the observation that proper roasting time was positively correlated with sweet taste (Ye et al., 2022).

To further understand the conversion of characteristic taste of Tieguanyin tea, the analysis on the dynamic changes of major flavor metabolites, including sugars, amino acids, flavonoids, xanthine alkaloids, and nucleotide, were performed. As presented in Fig. 5C, the abundance of key flavor metabolites changed dynamically after the drying step, in accordance with the alterations of taste attributes over Tieguanyin tea production. The contents of sugars, including fructose, galactose, trehalose, and maltose, increased after drying treatment, ascribing to the degradation of macromolecular substances such as starch (Mao et al., 2018).

The components associated with bitter or astringent tastes in teas are mainly composed of catechins, xanthine alkaloids, flavonol glycosides and several amino acids. The results of targeted UPLC analysis showed that the total contents of catechins in DT (130.78 mg g^−1^) were reduced by 20.19 % than in BF (163.87 mg g^−1^, Table S3). The contents of catechins decreased upon the harvest of fresh leaves was potentially attributed to the oxidation/polymerization reactions caused by endogenous enzymes during the ECP stage, and non-enzymatic oligomerization to high molecular weight complexes within thermal processing (Fan et al., 2016). A slight reduction of 7.15 % was observed in the contents of alkaloids in DT (27.79 mg/g) than in FL (29.93 mg/g) samples, mainly ascribing to the stable chemical property of caffein during the production process of oolong tea. The contents of most amino acids showed dynamic changes during oolong tea production process (Table S3). Most of the amino acids were increased over the ECP stage of Tieguanyin tea production, which is in line with our previous study, in which the increase of free amino acids was resulted from the degradation of proteins or peptides in response to the multi-stress encountered in ECP stage (Wu et al., 2022); additionally, the targeted UPLC analysis showed a sharp reduction in amino acid content in Tieguanyin tea after drying, the Maillard reaction occurred between the between aldoses and amino acids played an important role in the reduction of amino acids, contributing to the final quality of Tieguanyin tea (Song et al., 2023). However, further investigation on the inconsistence between the targeted and widely-targeted quantification of amino acid in Tieguanyin tea after drying is needed for a better profile of dynamic changes of these flavor related compounds. The dramatical decreasing of flavonol glycosides and increasing of flavanol aglycons such as kaempferol and quercetin observed after drying step (Fig. 5C) was caused by the de-glycosylation of flavonol glycosides under heating treatment (firing and drying), which is in line with the previous study (Fang et al., 2019). Taken together, the significant reduction in bitter or astringent components, and increase in sweet-associated compounds contributed to the mellow and rich flavor of Tieguanyin tea.

## 4 Conclusions

In this study, the metabolomic and transcriptomic profiles of non-volatiles were obtained from the samples at various processing steps of Tieguanyin tea production to reveal the metabolic pathways networking of flavor compounds. The joint transcriptome-metabolome analysis demonstrated that multiple stresses occurring during ECP stage induced alterations in key flavor compounds such as amino acids, sugars and flavonoids upon the harvest of fresh tea shoots. The long-term setting before firing is conducive to the conversion or accumulation of several key flavoring compounds. Furthermore, the thermal effect during the drying step improves sensory properties such as de-bittering, resulting in the mellow and rich flavor of Tieguanyin tea. Despite these achievements, the absent of targeted quantification of key taste compounds hindered a better understanding on the formation of characteristic flavor Tieguanyin tea, which needs to be further explored in the future. Nevertheless, our study provides a novel insight and framework to illustrate the biological process associated with flavor formation over the Tieguanyin tea production based on the complementary metabolomic and transcriptomic analyses.

## Declaration of Competing Interest

The authors declare that they have no known competing financial interests or personal relationships that could have appeared to influence the work reported in this paper.

## Data Availability

Data will be made available on request.
