# Supplementary material for: An integrated metabolomic and transcriptomic analysis reveals the dynamic changes of key metabolites and flavor formation over Tieguanyin oolong tea production
Source: Food Chem X. 2023 Oct 21;20:100952. doi: 10.1016/j.fochx.2023.100952 (PMC10618703; doi:10.1016/j.fochx.2023.100952)
Supplement: Supplementary data 3 [file mmc3.docx]

**Table S3 The contents of catechins, xanthine alkaloids and amino acids in different tea samples***

| Class | Compounds | Sample | | | |
| --- | --- | --- | --- | --- | --- |
|  |  | FL | WT | BF | DT |
| Catechins (mg/g) | (+)-Gallocatechin | 0.45±0.1 | 0.56±0.04 | 0.72±0.13 | 0.76±0.01 |
|  | (-)-Epi-gallocatechin | 5.19±0.44 | 5.78±0.21 | 10.73±0.34 | 6.23±0.71 |
|  | (+)-Catechin | 3.15±0.23 | 2.48±0.05 | 3.57±0.41 | 2.21±0.33 |
|  | (-)-Epi-catechin | 9.56±0.52 | 8.78±0.39 | 12.19±0.67 | 8.27±0.28 |
|  | (-)-Epi-gallocatechin gallate | 123.43±2.19 | 118.94±1.50 | 101.21±4.80 | 95.94±7.09 |
|  | (-)-Gallocatechin gallate | 1.08±0.04 | 1.43±0.05 | 0.91±0.02 | 1.54±0.05 |
|  | (-)-Epi-catechin gallate | 20.62±0.43 | 18.47±0.51 | 18.91±0.34 | 15.72±1.49 |
|  | (-)-Catechin gallate | 0.39±0.04 | 0.21±0.01 | 0.12±0.01 | 0.11±0.01 |
|  | Total contents | 163.87 | 156.65 | 148.36 | 130.78 |
| Xanthine alkaloids (mg/g) | Caffeine | 29.87±0.23 | 32.26±0.31 | 38.81±1.21 | 27.53±1.64 |
|  | Theophylline | 0.06±0.01 | 0.09±0.01 | 0.39±0.02 | 0.26±0.02 |
|  | Total contents | 29.93 | 32.35 | 39.20 | 27.79 |
| Amino acids (μg/g) | L-Isoleucine | 6.98±0.29 | 31.40±0.68 | 78.44±1.05 | 15.32±0.87 |
|  | L-Threonine | 134.47±3.42 | 244.46±5.81 | 299.01±7.37 | 85.32±3.24 |
|  | L-Valine | 8.74±0.52 | 46.15±0.88 | 95.61±2.37 | 10.25±0.23 |
|  | L-Asparagine | 27.42±0.97 | 83.57±2.20 | 215.01±4.43 | 17.93±0.18 |
|  | L-Proline | 24.05±0.88 | 98.04±3.69 | 117.51±5.39 | 49.74±2.39 |
|  | L-Histidine | 24.80±0.29 | 58.76±1.38 | 138.60±3.57 | 19.58±0.12 |
|  | L-Leucine | 7.14±0.05 | 57.44±2.27 | 111.39±3.38 | 48.58±1.09 |
|  | L-Tyrosine | 13.13±0.25 | 151.25±1.06 | 203.64±5.57 | 59.71±1.28 |
|  | L-Phenylalanine | 6.54±0.33 | 10.75±0.39 | 57.92±1.28 | 10.93±0.34 |
|  | L-Tryptophan | 40.24±2.85 | 92.56±4.71 | 242.52±6.57 | 38.13±0.97 |
|  | L-Methionine | 2.86±0.12 | 2.28±0.18 | 4.77±0.37 | 1.31±0.08 |
|  | L-Serine | 281.20±9.37 | 463.07±17.37 | 469.45±20.73 | 306.15±12.33 |
|  | L-Glutamine | 2413.94±923.56 | 770.38±50.25 | 1074.23±93.81 | 388.72±42.39 |
|  | L-Arginine | 79.93±3.74 | 47.54±2.89 | 50.83±2.38 | 30.74±2.10 |
|  | L-Aspartate | 770.90±35.26 | 1163.95±50.73 | 1485.28±70.28 | 721.58±40.85 |
|  | L-Lysine | 17.01±0.89 | 94.81±6.75 | 139.15±8.34 | 40.21±1.24 |
|  | L-Alanine | 155.07±5.25 | 141.72±4.23 | 141.27±7.15 | 60.57±3.52 |
|  | L-Glycine | 31.47±1.27 | 8.33±0.36 | 9.82±0.84 | 2.36±0.16 |
|  | L-Glutamic acid | 997.79±49.20 | 938.46±62.78 | 994.83±60.17 | 310.84±15.78 |
|  | L-Theanine | 8651.59±728.93 | 9627.06±926.34 | 8311.67±509.23 | 4936.28±323.26 |
|  | γ-Aminobutyric Acid | 662.04±40.49 | 1876.41±73.15 | 1448.84±79.17 | 761.72±50.72 |
|  | Total content | 14357.31 | 16008.39 | 15689.79 | 7915.97 |

*: FL, WT, T3 and DT represent fresh leaf, leaf after withering, leaf after 3rd turning-over and dried tea, respectively.
